# Supplementary material for: Mesoscale simulation of biomembranes with FreeDTS
Source: Nat Commun. 2024 Jan 16;15:548. doi: 10.1038/s41467-024-44819-w (PMC10792169; doi:10.1038/s41467-024-44819-w)
Supplement: Supplementary file 1 — Supplementary Information [file 41467_2024_44819_MOESM1_ESM.pdf]

Supplementary Materials
Mesoscale simulation of biomembranes with FreeDTS

Weria Pezeshkian[1]* and John H. Ipsen[2]

[1]Niels Bohr International Academy, Niels Bohr Institute, University of Copenhagen, Blegdamsvej 17, 2100 Copenhagen, DENMARK.
[2] MEMPHYS/PhyLife, Department of Physics, Chemistry and Pharmacy (FKF), University of Southern Denmark, Campusvej 55, 5230 Odense M, Denmark.

*Corresponding author: weria.pezeshkian@nbi.ku.dk

Table of Contents

Supplementary Note 1 .... ***2***

Converting $ldts$ to a physical unit **2**

Supplementary Note 2 .... ***2***

Discrete shape operator, principal directions, and curvatures **2**

Supplementary Note 3 .... ***3***

Constant tension algorithm (dynamic box) **3**

Supplementary Note 4 .... ***3***

Osmotic pressure **3**

Supplementary Note 5 .... ***4***

System evolution **4**

Supplementary Note 6 .... ***4***

Benchmark: performance of the code for the Monte Carlo moves **4**

Supplementary Tables ***5***

Supplementary Figures ***5***

Supplementary References ***11***

Supplementary Note 1

Converting l_{dts} to a physical unit

While it is possible and very common to explore membrane behaviors in terms of relative length scales (in the unit of l_{dts}) [1, 2] often it is more appealing to convert this length unit (l_{dts}) to a physical unit in particular if we are focusing on a specific real system. Previously we have done this for Shiga toxin, Annexin 4 and simple vesicles [3, 4]. However, this conversion scheme is not unique and can be adopted according to the phenomenon we are interested in. We briefly discuss this topic below and refer interested readers to the original articles [3-6].

For system containing proteins: To convert l_{dts} to a physical unit e.g., nm, we assume that the area of a vertex which is $\sqrt{3}/2l_{dts}^2$ is equal to the area is equal to the area of the smallest circle wrapping the protein (πR^2) [3, 6]. Therefore,

$$l_{dts} \sim 1.9\, R \qquad (11)$$

For pure bilayer: When two faces of a membrane approach each other, within nm distance, they repel each other through hydration and membrane protrusion forces that are below the DTS resolution. We account for these forces by treating them as infinite repulsion by restricting different segments of a membrane surface from approaching a distance at which these forces are effective (0.7 nm). Thus, l_{dts} is the bilayer thickness plus a distance in which hydration and membrane protrusion forces are effective ($\sim 0.7nm$), leading to $l_{dts} \sim 4.5nm$. Note, for membranes with small deformations, $l_{dts} > 4.5nm$ nm can also be chosen. However, for highly deformed membranes, this choice implies that some realistic configurations of the membrane have been eliminated[4].

Supplementary Note 2

Discrete shape operator, principal directions, and curvatures

Vertex normal and area: On each vertex, a normal ($\hat{\boldsymbol{n}}_v$) and an area associated with the vertex (A_v) are defined as

$$A_v = \frac{1}{3}\sum_{T(R)} A_T \qquad (1)$$

$$\hat{\boldsymbol{n}}_v = \frac{\sum_{T(R)} A_T \hat{\boldsymbol{n}}_T}{\left|\sum_{T(R)} A_T \hat{\boldsymbol{n}}_T\right|} \qquad (2)$$

Where $\sum_{T(R)} \dots$ indicates that the sum is over faces (triangles) in a ring around the vertex. A_T and $\hat{\boldsymbol{n}}_T$ are the triangle area and normal.

Edge normal: A normal associated with an edge ($\hat{\boldsymbol{n}}_e$) is obtained using normals of two triangles sharing the edge ($\hat{\boldsymbol{n}}_{T1}, \hat{\boldsymbol{n}}_{T2}$) as

$$\hat{\boldsymbol{n}}_e = \frac{\hat{\boldsymbol{n}}_{T1} + \hat{\boldsymbol{n}}_{T2}}{|\hat{\boldsymbol{n}}_{T1} + \hat{\boldsymbol{n}}_{T2}|} \qquad (3)$$

Shape operator on an edge: The shape operator on an edge $\boldsymbol{e}$ is be obtained as

$$\overleftrightarrow{\boldsymbol{S}}_e = H_e(\vec{\boldsymbol{r}}_e \times \hat{\boldsymbol{n}}_e)\otimes(\vec{\boldsymbol{r}}_e \times \hat{\boldsymbol{n}}_e) \qquad (4)$$

Where $H_e = 2|\vec{r}_e|\cos\frac{\Phi}{2}$ and Φ is the signed dihedral angle between the two triangles ($\hat{\boldsymbol{n}}_{T1}$ and $\hat{\boldsymbol{n}}_{T2}$) sharing the edge e and $\vec{r}_e$ is the unit vector along edge $\boldsymbol{e}$.

Shape operator on a vertex: The shape operator on each vertex ($\overleftrightarrow{\boldsymbol{S}}_v$) is obtained as

$$\overleftrightarrow{\mathbf{S}}_{\boldsymbol{v}} = \frac{\mathbf{1}}{A_v}\sum_{e(R)}(\hat{\boldsymbol{n}}_v.\hat{\boldsymbol{n}}_e)\overleftrightarrow{\mathbf{P}}_{\boldsymbol{v}}^{\dagger}\overleftrightarrow{\mathbf{S}}_{\boldsymbol{e}}\overleftrightarrow{\mathbf{P}}_{\boldsymbol{v}} \quad (5)$$

Where $\sum_{e(R)}$ ... indicates that the sum is over edges in a ring around the vertex and $\overleftrightarrow{\mathbf{P}}_{\boldsymbol{v}}$ is a projection operator that projects geometrical objects on the tangent plane of the vertex v that is given by

$$\overleftrightarrow{\mathbf{P}}_{\boldsymbol{v}} = \mathbb{I} - \hat{\boldsymbol{n}}_v \otimes \hat{\boldsymbol{n}}_v \qquad (6)$$

Principal curvatures and directions: Eigenvector $\overleftrightarrow{\mathbf{S}}_v$ are vertex normal, and principal directions $(\hat{\mathbf{T}}_1, \hat{\mathbf{T}}_2, \hat{\boldsymbol{n}}_v)$ while eigenvalues are c_1, c_2 and zero $(c_1, c_2, 0)$.

Supplementary Note 3

Constant tension algorithm (dynamic box)

To simulate a membrane with constant frame tension (τ), there is an extra move to change the membrane frame area. Let's assume our framed membrane has a projected area A_p. on XY plane in a rectangular box $(Lx \quad Ly \quad Lz)$ with periodic boundary conditions in all three directions. To each Monte Carlo Sweep (MCS) with a probability of $P = 1/(N_v t_0)$ the bilayer frame area is updated and with probability of $1 - P$, other trial moves are performed. To update the projected area: first, the box sides will be updated to $Lx \rightarrow Lx + \Delta Lx$ and $Ly \rightarrow Ly + \Delta Ly$ in which ΔLx is chosen randomly in the interval $[\ 0.04 d_{dts}, 0.04 d_{dts}]$ and $\Delta Ly = \Delta Lx(Lx/Ly)$. The X, Y coordinates at each vertex are then updated to $X \rightarrow (1 + \Delta Lx/Lx)X$ and $Y \rightarrow (1 + \Delta Ly/Ly)Y$. To accept or reject the move, Metropolis algorithm with the acceptance probability (P_{acc}) at temperature T is performed, where:

$$P_{acc} = \min\left[1, \left[\frac{A_{p-new}}{A_{p-old}}\right]^{N_v} \exp\left(\tau \Delta A_p - \Delta E\right)\right] \qquad (7)$$

Note: We have set $k_B T = 1$, therefore all the energy unit in FreeDTS is in $k_B T$.

Supplementary Note 4

Osmotic pressure

To model the effect of osmotic pressure, FreeDTS can also apply an algorithm based on the Jacobus van 't Hoff equation, $\Pi = icRT$, where i is van 't Hoff index, c is the molar concentration of solute, R is the ideal gas constant, and T is the temperature. For a vesicle system, we define, V_{ini} as the initial volume of the vesicle and effective compartment concertation of solute as $\bar{c}_j = \sum_n i_n c_n$, where j is either inside (at V_{ini}), or outside and the summation run over all the solute types in the j compartment. Therefore, we have

$$\Delta\Pi = RT\left(\frac{V_{ini}\bar{c}_{in}}{V} - \bar{c}_{out}\right) \quad (8)$$

Energy associated with changes of the vesicle volume from V_{ini} to V will be obtained as

$$\Delta E_{osmos}(V) = -RT\int_{V_{ini}}^{V} \Delta\Pi dV = -RT\left[\bar{c}_{in}V_{ini}\ln\frac{V}{V_{ini}} - \bar{c}_{out}(V - V_{ini})\right] \quad (9)$$

FreeDTS can directly use equation 3 to apply osmotic pressure difference. However, in most cases, this will be equivalent to the equation 3 in the main manuscript since

$$\Delta E_{osmos}(V) = RT\bar{c}_{out}V_{\mathrm{eq}}\left\{\frac{1}{2}\left(\frac{V - V_{\mathrm{eq}}}{V_{\mathrm{eq}}}\right)^2 + \left[\sum_{i=3}^{\infty}\left(\frac{V_{\mathrm{eq}} - V}{iV_{\mathrm{eq}}}\right)^i\right]\right\} \quad (10)$$

Where $\bar{c}_{out}V_{\mathrm{eq}} = \bar{c}_{in}V_{ini}$ and $K = RT\bar{c}_{out}V_{eq}$. In typical experiments, $\bar{c}_{out}$ and $\bar{c}_{in}$ are in the order of $\sim 10mM$ and vesicle volume is $V \sim \mu m^3$, making, $K \sim 10^5 - 10^6 kT$ that is much larger than bending energy

associated with vesicle deformations at this scale ($4\pi\kappa \sim 10^2 - 10^3\ kT$). Therefore, only the first term of equation 4 will be relevant and the vesicles will adopt to $V = V_{\text{eq}} = \frac{\bar{c}_{in} V_{ini}}{\bar{c}_{out}}$.

Supplementary Note 5

System evolution

System evolution and the equilibrium properties of the membranes are evaluated by standard Monte Carlo sampling of Boltzmann's probability distribution. We have set $k_B T = 1$, therefore all the energy unit in FreeDTS is in $k_B T$. Every Monte Carlo move consists of,

1) N_v vertex positional updates.
2) N_e Alexander moves: a trial to flip the mutual link between two neighboring triangles.
3) N_i inclusion moves where N_i is the number of the inclusions in the system. An inclusion move can be either rotation or a Kawasaki move.

In FreeDTS there are two options to perform these moves.

1) $N_v + N_e + N_i$ sweep attempts is one step. At each attempt, with probability of $N_v/(N_v + N_e + N_i)$ the selected move is vertex position update; with probability of $N_e/(N_v + N_e + N_i)$ the selected move is Alexander moves and with probability of $N_i/(N_v + N_e + N_i)$ the selected move is inclusion moves.
2) In one step N_v vertex update, N_e Alexander moves and N_i inclusion moves will be performed.

If a system is coupled to the tension controlling scheme, then there will be also one trial move to change the box size with the a given probability (see Supplementary Note 3). There is also a possibility to activate parallel tempering algorithm. This will run multiple replicas at different temperature using OpenMP parallelization and after certain steps (defined by the user) it exchange the conformation of the replicas/

Supplementary Note 6

Benchmark: performance of the code for the Monte Carlo moves

In order to test the performance of the code, we measured the time elapsed for each move on four different systems. These systems are chosen to consist of the most (computationally) simple system (system 1, a closed triangulated surface) up to the most complex system (system 4: a periodic flat membrane fully decorated with the most complicated inclusions). The results were collected on a ThinkStation P620, model name AMD Ryzen Threadripper PRO 3995WX 64-Cores. Note, while the system contains 64 cores, the simulations were performed on a single core with a core frequency of "CPU max GHz 4.308 and CPU min GHz 2.200". In Supplementary Table 2, the time elapsed between these moves is shown (reported for 1000 moves). As the number of calculations and, therefore, the duration of each move is dependent upon the results of the move (accepted or rejected and what is the cause of rejection), we monitored the time elapsed for each move in Supplementary Figures 9-12. In these figures, only the uppermost lines are the accepted moves. Below zero is a rejected move and the simulation results presented in the main manuscript was obtained by simulations of $10^6 - 10^7$ steps which is resulting in 1-10 days of simulations on the described workstation. Please note a set of $N_v + N_e + N_i$ sweep attempts is called one MC step (see Supplementary Note 5).

Supplementary Tables

System	Area per vertex
Vesicle (i)	1.545 ± 0.008
Tensionless flat membrane (ii)	1.548 ± 0.007
Flat membrane with constant AP (iii)	1.540 ± 0.003
Tensionless flat membrane with inclusions (iv)	1.547 ± 0.007
Tensionless flat membrane between two walls (v)	1.546 ± 0.006

Supplementary Table 1: Area per vertex remains constant for membrane simulations in different conditions. Also see Supplementary Figure 6.

	Time Per 1000 Moves [millisecond]
Link flip move (alexander move)	0.45-0.95
Vertex position update move	24-32
Inclusion moves	1-1.1
Box size update move	2.5 (per vertex)

Supplementary Table 2: The range of expected time elapse for each Monte Carlo move.

Supplementary Figures

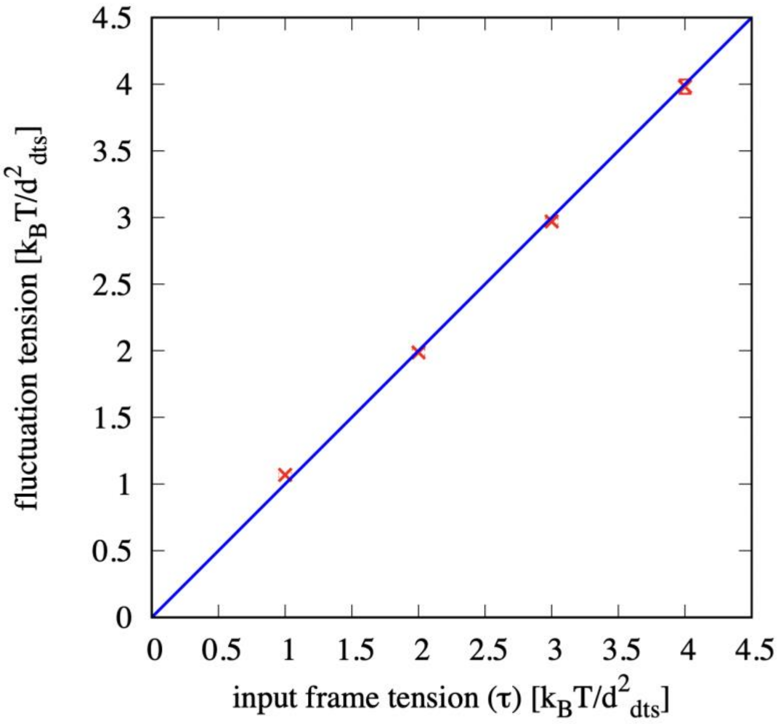

Supplementary Figure 1: The membrane tension determined by the undulation spectrum as a function of the input frame tension. These results indicate that they are equal. Source data are provided as a Source Data file.

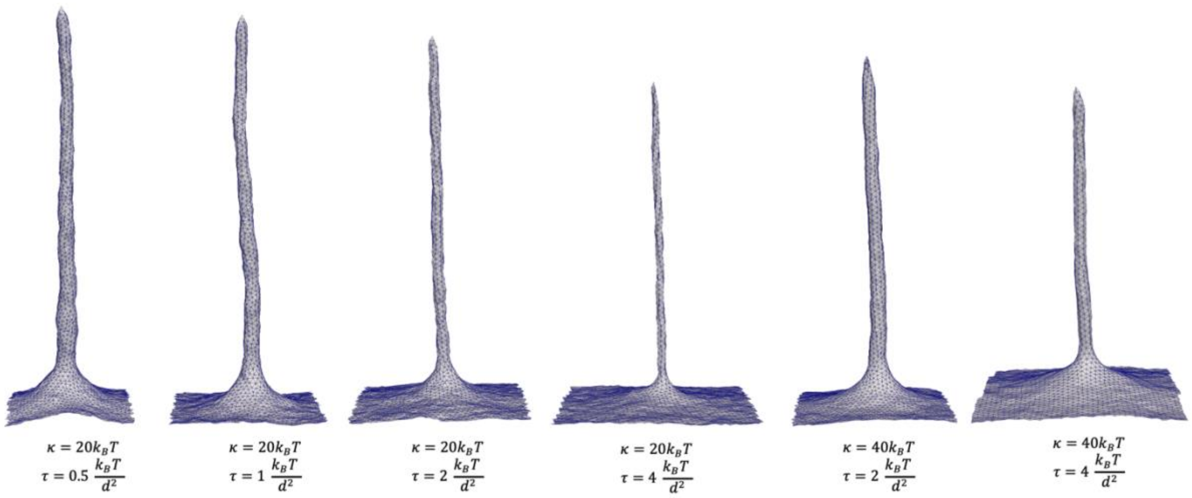

Supplementary Figure 2: Pulled Tubes of different radii. By changing membrane tension or bending rigidity, tubes with different radii will form.

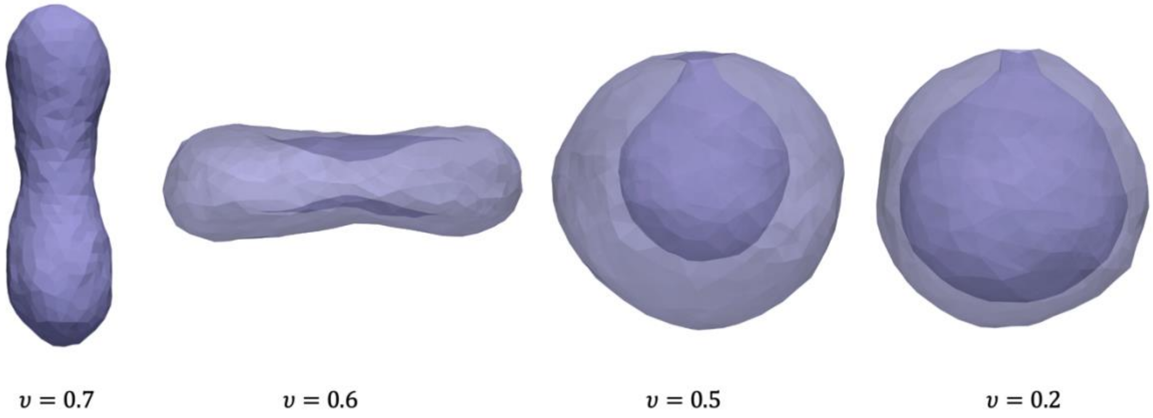

Supplementary Figure 3: Transition of a spherical vesicle from prolate-to-oblate and oblate-to-stomatocyte by volume reduction.

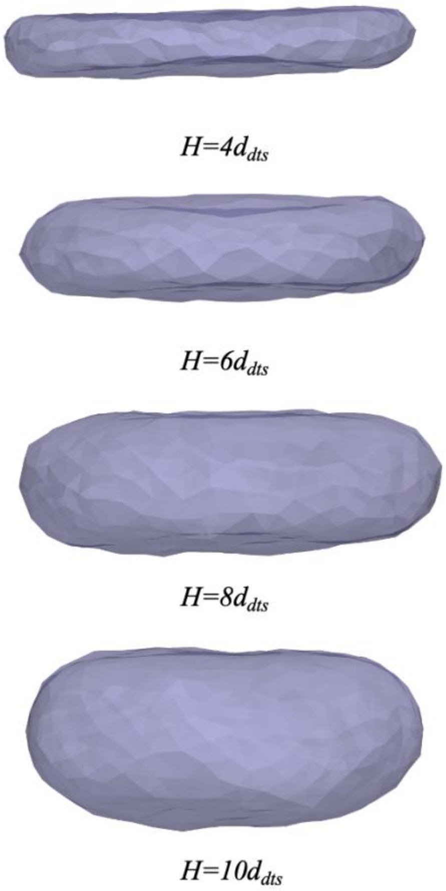

Supplementary Figure 4: A vesicle sandwiched between two confining walls in the Z direction for different distance between the walls.

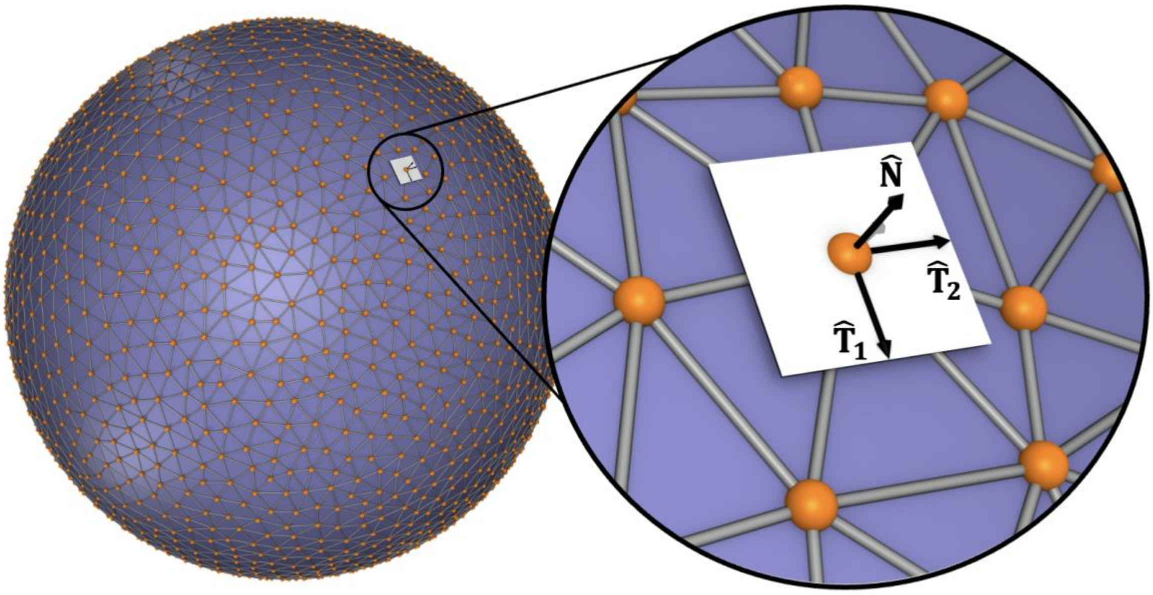

Supplementary Figure 5: A regular triangulated surface containing N_v vertices, N_e edges and N_T triangles. Using a set of discrete gematrical operations we obtain, principal curvatures (c_1, c_2), and principal directions $(\widehat{\mathbf{T}}_1, \widehat{\mathbf{T}}_2)$, and a surface normal $(\widehat{\mathbf{N}})$ on each vertex.

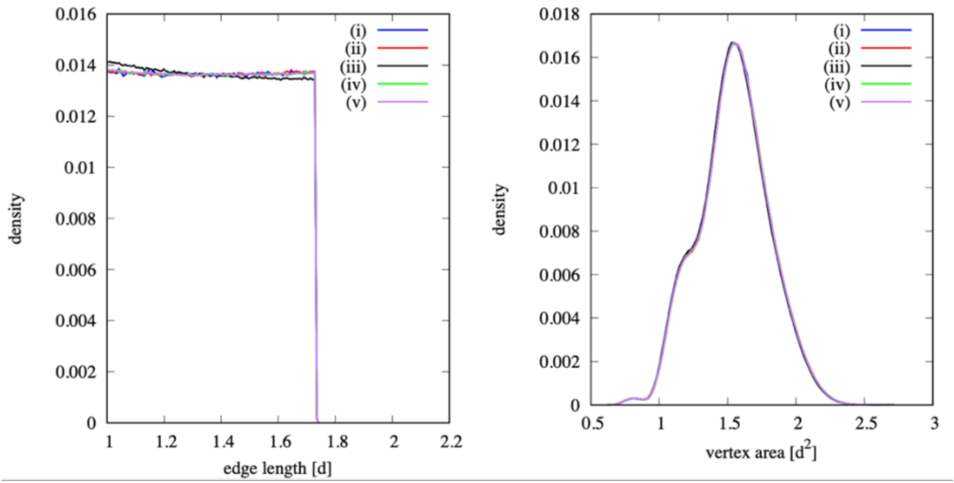

Supplementary Figure 6: Distributions for the systems shown in the Supplementary Table 1. Source data are provided as a Source Data file.

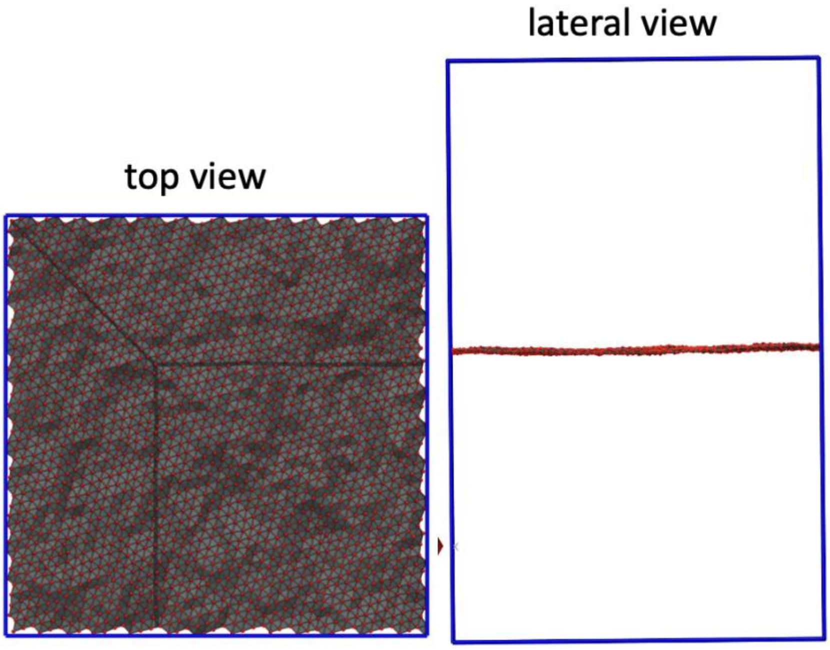

Supplementary Figure 7: Two different views of a triangulated surface in a periodic box. Note, the vertices along each edge are connected with those on the opposite side through the periodic box. This connectivity cannot be visualized.

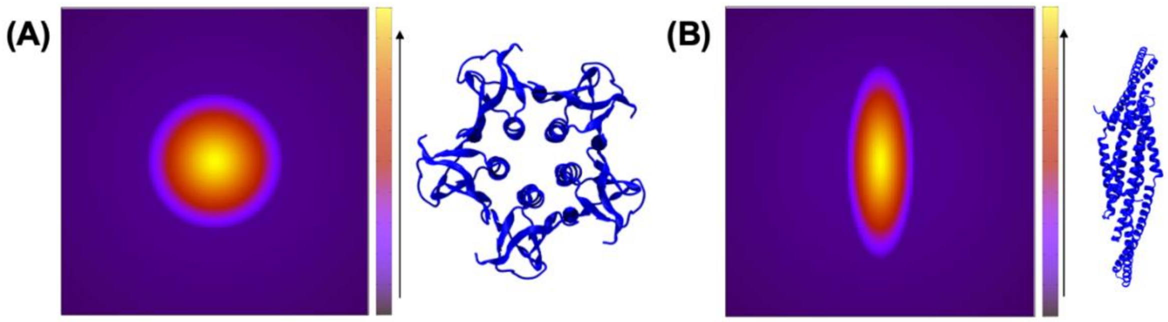

Supplementary Figure 8: Two different protein types in FreeDTS. (A) Protein type 1, in which its interaction with the membrane modifies the membrane in a symmetric (or almost symmetric) way (B)

Protein type 1, in which its interaction with the membrane modifies the membrane in a asymmetric way.

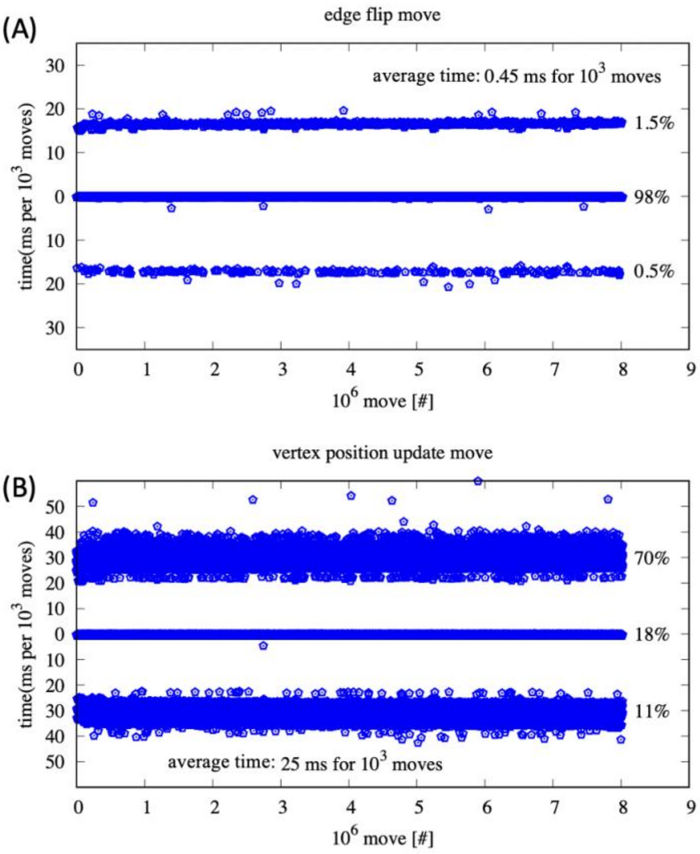

Supplementary Figure 9: A spherical triangulated mesh containing 802 vertices. Only the uppermost line are the accepted moves. (A) Link flip attempts. (B) vertex position update. Source data are provided as a Source Data file.

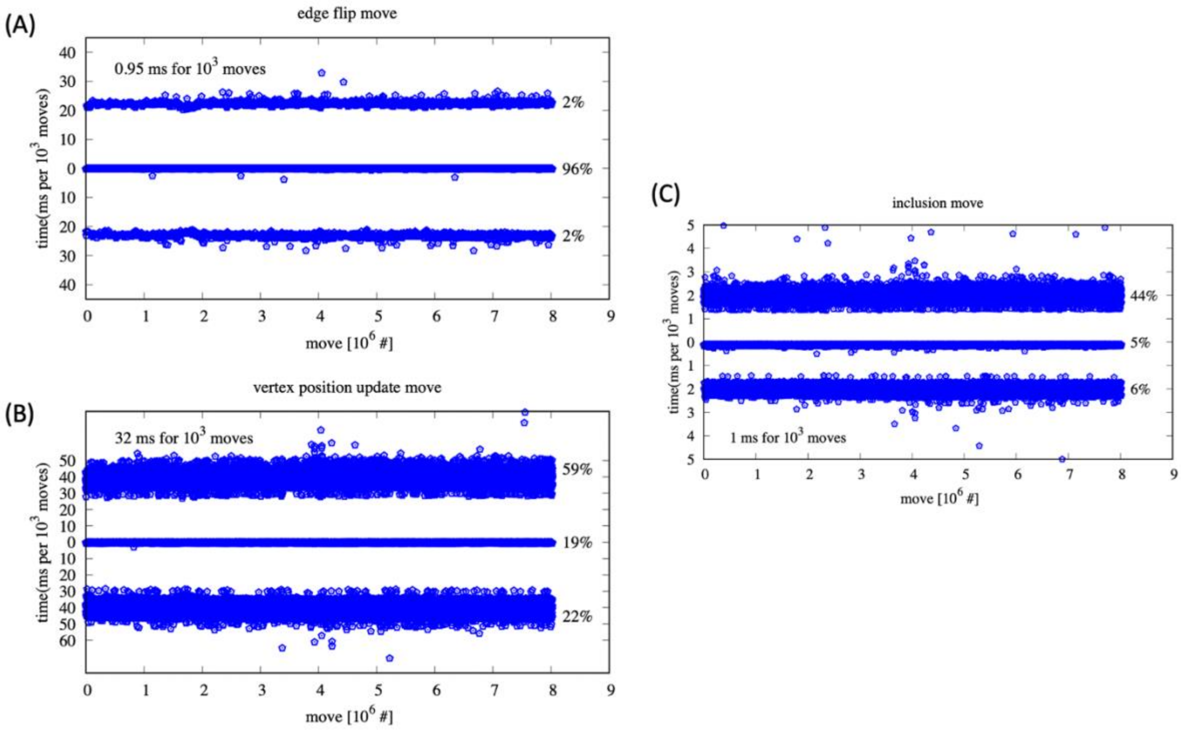

Supplementary Figure 10: A spherical triangulated mesh containing 802 vertices and fully covered by inclusions. Only the uppermost line are the accepted moves. (A) Link flip attempts. (B) vertex position update. (C) inclusion moves. Source data are provided as a Source Data file.

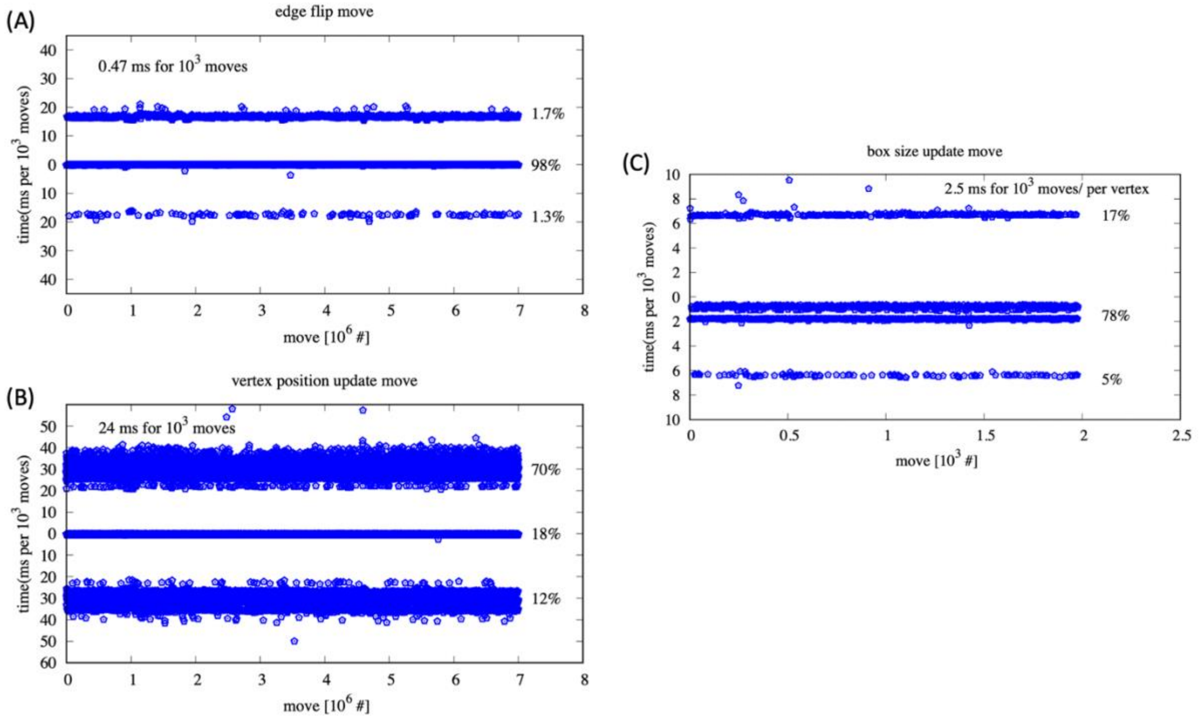

Supplementary Figure 11: A flat period triangulated mesh containing 700 vertices coupled with the tension controlling algorithm. Only the uppermost line are the accepted moves. (A) Link flip attempts. (B) vertex position update. (C) box size update move. Source data are provided as a Source Data file.

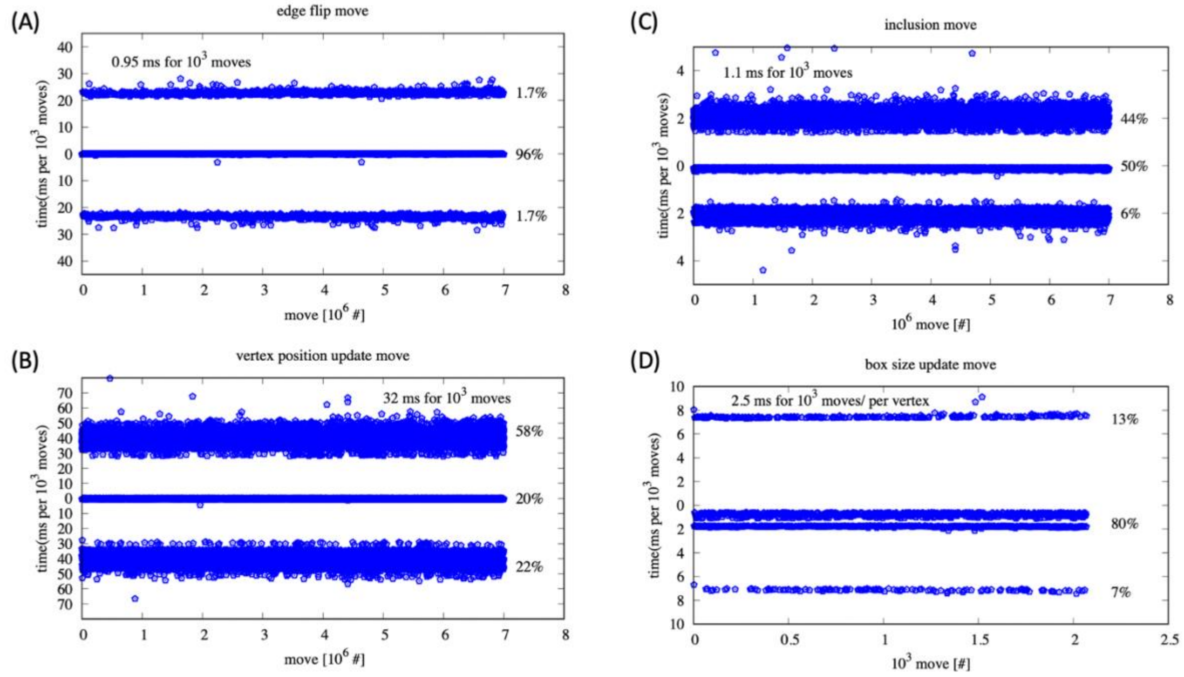

Supplementary Figure 12: A flat period triangulated mesh containing 700 vertices coupled with the tension controlling algorithm. Only the uppermost line are the accepted moves. (A) Link flip attempts. (B) vertex position update move. (C) inclusion moves. (D) box size update move. Source data are provided as a Source Data file.

Supplementary References

[1] W. Pezeshkian, J.H. Ipsen, Creasing of flexible membranes at vanishing tension, Physical Review E 103(4) (2021) L041001.
[2] W. Pezeshkian, J.H. Ipsen, Fluctuations and conformational stability of a membrane patch with curvature inducing inclusions, Soft Matter 15(48) (2019) 9974-9981.
[3] C.D. Florentsen, A. Kamp-Sonne, G. Moreno-Pescador, W. Pezeshkian, A.A. Hakami Zanjani, H. Khandelia, J. Nylandsted, P.M. Bendix, Annexin A4 trimers are recruited by high membrane curvatures in giant plasma membrane vesicles, Soft Matter 17(2) (2021) 308-318.
[4] W. Pezeshkian, M. Konig, T.A. Wassenaar, S.J. Marrink, Backmapping triangulated surfaces to coarse-grained membrane models, Nat Commun 11(1) (2020) 2296.
[5] W. Pezeshkian, A.G. Hansen, L. Johannes, H. Khandelia, J.C. Shillcock, P.B. Kumar, J.H. Ipsen, Membrane invagination induced by Shiga toxin B-subunit: from molecular structure to tube formation, Soft Matter 12(23) (2016) 5164-71.
[6] W. Pezeshkian, M. Konig, S.J. Marrink, J.H. Ipsen, A Multi-Scale Approach to Membrane Remodeling Processes, Front Mol Biosci 6 (2019) 59.
