# Supplementary material for: Mesoscale simulation of biomembranes with FreeDTS
Source: Nat Commun. 2024 Jan 16;15:548. doi: 10.1038/s41467-024-44819-w (PMC10792169; doi:10.1038/s41467-024-44819-w)
Supplement: Supplementary file 3 — Description of additional supplementary files [file 41467_2024_44819_MOESM3_ESM.pdf]

Description of additional supplementary files

Supplementary Movie 1 : Pulling a nanotube from a membrane patch with periodic boundary conditions by exerting a harmonic force on a single vertex.
